# Germline Variants in Cancer Genes from Young Breast Cancer Mexican Patients

**DOI:** 10.3390/cancers14071647

**Published:** 2022-03-24

**Authors:** Liliana Gómez-Flores-Ramos, Angélica Leticia Barraza-Arellano, Alejandro Mohar, Miguel Trujillo-Martínez, Lizbeth Grimaldo, Rocío Ortiz-Lopez, Víctor Treviño

**Affiliations:** 1 CONACYT/Center for Population Health Research, National Institute of Public Health, Universidad No. 655, Cuernavaca 62100, Morelos, Mexico; liliana.gomez@insp.mx (L.G.-F.-R.); cisp73@insp.mx (L.G.); 2 School of Medicine, Tecnologico de Monterrey, Morones Prieto Av 3000, Los Doctores, Monterrey 64710, Nuevo Leon, Mexico; barraza.leticia@exatec.tec.mx (A.L.B.-A.); rortizl@tec.mx (R.O.-L.); 3 Unidad de Investigación Biomédica en Cáncer, Instituto de Investigaciones Biomédicas, Universidad Nacional Autónoma de México, Dirección de Investigación, Instituto Nacional de Cancerología, Av. San Fernando #22, Col. Sección XVI, Delegación Tlalpan, Mexico City 14080, Mexico; mohar@iibiomedicas.unam.mx; 4 Instituto Mexicano del Seguro Social, Hospital General de Zona con Medicina Familiar No. 7, Cuautla 62780, Morelos, Mexico; miguel.trujillom@imss.gob.mx; 5 The Institute for Obesity Research, Tecnologico de Monterrey, Eugenio Garza Sada Av 2501, Monterrey 64849, Nuevo Leon, Mexico

**Keywords:** breast cancer, variant annotation, variant pathogenicity, cancer predisposition, bioinformatic pipeline, cancer genes

## Abstract

**Simple Summary:**

Young, Mexican women are more susceptible to breast cancer compared to other populations. However, studies on germline young, Mexican women are scarce and limited to a panel of 143 genes. This is partially due to the lack of gene annotation tools and difficulties in determining the causal genes in understudied populations. Here, we used whole exome sequencing combined with a powerful annotation tool to analyze 862 cancer genes in 115 young, Mexican women diagnosed with breast cancer. Our results showed well-known genes and many barely reported variants in our population. Moreover, we were able to assign candidate causal genes to 34% of patients, surpassing previous studies. These results suggest that deeper bioinformatic analyses could inform medical decisions to improve diagnosis, treatment, and life expectancy in young women with breast cancer.

**Abstract:**

Breast cancer (BC) is one of the most frequent cancer types in women worldwide. About 7% is diagnosed in young women (YBC) less than 40 years old. In Mexico, however, YBC reaches 15% suggesting a higher genetic susceptibility. There have been some reports of germline variants in YBC across the world. However, there is only one report from a Mexican population, which is not restricted by age and limited to a panel of 143 genes resulting in 15% of patients carrying putatively pathogenic variants. Nevertheless, expanding the analysis to whole exome involves using more complex tools to determine which genes and variants could be pathogenic. We used germline whole exome sequencing combined with the PeCanPie tool to analyze exome variants in 115 YBC patients. Our results showed that we were able to identify 49 high likely pathogenic variants involving 40 genes on 34% of patients. We noted many genes already reported in BC and YBC worldwide, such as *BRCA1*, *BRCA2*, *ATM*, *CHEK2*, *PALB2*, and *POLQ*, but also others not commonly reported in YBC in Latin America, such as *CLTCL1*, *DDX3X*, *ERCC6*, *FANCE*, and *NFKBIE*. We show further supporting and controversial evidence for some of these genes. We conclude that exome sequencing combined with robust annotation tools and further analysis, can identify more genes and more patients affected by germline mutations in cancer.

## 1. Introduction

Breast cancer (BC) is one of the most frequent types of cancer among women; 2.3 million cases were reported in 2020, causing more than 680,000 deaths globally [1]. The BC incidence increases with age. The majority of the diagnosed patients are 40 years and older, reaching 89%: 4% between 39 and 35, 5% less than 35, and 1.75% in women under 30 (https://gco.iarc.fr/ (accessed on 1 December 2021), [1]). In Mexico, BC is the first cause of death among women diagnosed with a malignant neoplasia [1], with an estimate of 26% of cancer-related deaths. Notably, the incidence of breast cancer in young women (YBC) in Mexico reached 15% in 2020, which is considerably higher than worldwide [1,2].

It has been observed that clinical outcomes and tumor biology in young patients are different from older women [3,4]. Tumors in young women are more likely to be of higher histological grade and are usually classified as estrogen receptor (ER) and progesterone negative receptors [5]. These patients typically present local recurrences to be diagnosed at an advanced stage and have inferior 5-year survival compared to the older premenopausal counterparts [5]. Moreover, YBC tends to present a more aggressive subtype such as triple-negative or HER2+ [6].

In some populations, up to 24% of the hereditary BC is linked to germline mutations in *BRCA1/2* specific genes [7], and the prevalence may reach 14% in patients not showing familial history [7]. Mutations in these genes have a lifetime risk of developing BC up to 65% [8]. Other genes with germline mutations had been reported to confer moderate cancer risk, such as *ATM*, *CHECK2*, and *PALB2*, which are also associated with other cancers [8,9,10].

In addition to *BRCA1*, *BRCA2*, *ATM*, *CHEK2*, and *PALB2*, germline mutations in Latin America have been reported for *CDH1*, *NBN*, *NF1*, *TP53*, *MLH1*, *BRIP1*, *MSH2*, *MSH6*, and *PMS2* in populations from Chile, Brazil, Colombia, and México [11]. In Mexico, a study in hereditary breast and ovarian cancer, which used a panel of 143 genes, found 21 germline mutations in *BRCA1* and *BRCA2*, while another 19 genes showed 1 or 2 mutations, including the *FANC*(*I/B/C/L/M*) gene family accounting for 6 mutations [12]. Importantly, the above study found only 15% of patients (46 of 300) showing a germline mutation in at least one of the 143 panel genes. The low detection rate highlights the need to interrogate more genes in the population known to carry familial susceptibility.

The sequencing of gene panels has been a cost-efficient tool to determine the prevalence of specific genes among cancer populations [13,14,15,16]. However, more genes need to be studied to determine pathogenicity in most patients, for example, by whole exome sequencing. One issue is that pathogenicity is challenging to assess in not well-known or reported genes without further functional assays. Fortunately, extensive sequencing efforts, such as the 1000 genomes project [17] and the gnomAD (accessed on 1 December 2021) [18], provide databases of human variation among a relatively healthy population that help remove many common variants. In addition, other databases, such as ClinVar (accessed on 1 December 2021), help to mark pathogenic variants [19]. Still, massive sequencing generates several unseen variants increasing the need to explore methodologies to identify possible causal variants. In this context, Pediatric Cancer Variant Pathogenicity Information Exchange (PeCanPIE, accessed on 1 December 2021) is a web-based tool that integrates many sources of information supported by the guidelines from the American College of Medical Genetics and Genomics (ACMG) that are useful to identify interesting candidate variants quickly [20]. PeCanPIE uses a variant categorization for putative pathogenicity based on a “medal ceremony” concept of four levels. The *Gold* category is assigned for highly likely pathogenic truncating or splicing variants, whose genes are already found in pathogenic databases and whose variants are rare among healthy populations. *Silver* variants are in-frame, indels, and truncations in non-cancer genes or predicted to be damaging and matching pathogenic databases. *Bronze* variants are those whose effects are predicted to be tolerated. An *unknown* label is assigned otherwise.

To determine the possible etiology of BC in young women, in this study, we performed whole exome sequencing followed by the PeCanPie bioinformatic analysis of 115 young patients diagnosed with BC and focused on known 862 cancer genes where pathology has already been associated with diseases. Briefly, we obtained 49 variants classified as *Gold* resulting in more than 20 genes that have not been reported previously in LA BC. In addition, 106 variants were classified as Silver.

## 2. Materials and Methods

### 2.1. Patients

We included 115 patients from the National Cancer Institute of Mexico (INCAN, Instituto Nacional de Cancerología) diagnosed at 40 years old or younger with histopathological confirmation of BC. Patients were recruited between September 2015 and December 2017. Medical records and electronic files with detailed clinical and sociodemographic information were obtained from the INCAN. Trained nurses obtained blood samples after patients received genetic counseling and signed the informed consent.

### 2.2. Ethical Considerations

Regulatory approval was obtained from the INCAN Research and Ethics Committee (Approval ID CEI/1123/16). Genetic counseling and information about potential germline findings were provided to patients in addition to assurance of patient confidentiality and relevant information concerning the project, sample management, and DNA shipment for analysis to the National Cancer Institute (NCI), USA. Based on the recommendations provided by the American College of Medical Genetics, hereditary cancer diagnostic variants in their DNA were reported to the patients.

### 2.3. Sample and Panel Library Preparation for Sequencing

The DNA was extracted from peripheral leukocytes using the Wizard Genomic DNA Purification Kit (Promega Corporation, Madison, WI, USA), following the manufacturer’s instructions. The resulting DNA was purified using Agencourt AMPure XP reagent (Beckman Coulter Inc., Brea, CA, USA) following the manufacturer’s protocol. In addition, an adapter-ligated library was prepared using the KAPA HyperPlus Kit (KAPA Biosystems, Wilmington, MA, USA) with NEXTflex^TM^ DNA Barcoded Adapters (Perkin Elmer Waltham, MA, USA), according to the KAPA-provided protocol.

### 2.4. Sequencing

The deep whole exome sequencing was performed at the Laboratory of Translational Genomics, Division of Cancer Epidemiology and Genetics (NCI, Rockville, MD, USA). The GRCh37 (hg19) genome assembly was used for genome mapping reference. The DNA sequencing was performed using the Illumina HiSeq 2000 sequencer for 2 × 100-pb paired-end cartridge (Illumina, San Diego, CA, USA). The sequencing included regulatory, splicing, and 3′ and 5′ UTR regions.

### 2.5. Variant Calling

The reads were aligned using Novoalign software (v3.00.05, Novocraft Technologies Sdn Bhd, Petaling Jaya Selangor, Malaysia). Duplicate reads were removed using MarkDuplicates from Picard Software (v1.126, Broad Institute, Cambridge, MA, USA). For variant calling, the pipeline involved RealignerTargetCreator, IndelReligner, BaseRecalibrator, UnifiedGenotyper, and HaplotyperCaller tools from GATK (v4.1.3.0, Broad Institute, Cambridge, MA, USA). Variants that failed to pass the pipeline control metrics (CScorefilter) had a read depth minor to 10, ABHet (reference to alternate reads ratio) <0.2 or >0.8 were excluded for the analysis. In addition, all variants were filtered using popmaxfreq <0.01.

Before variant categorization, variants were filtered to remove those that do not show at least 10 reads in the alternate allele or that the minor allelic fraction was lower than 0.25.

### 2.6. Variant Categorization

The obtained variant calling files were analyzed using PeCanPIE, which classified the variants into three categories: *Gold*, *Silver*, and *Bronze* [20]. The variants were assigned as *Gold* if it is a truncating variant, including splicing, or a loss-of-function associated with a reported disease documented in ClinVar with at least two starts, and at least one of the following databases: IARC: Tp53, ASU: TERT, ARUP: Ret, BIC, PCFP, and COSMIC. The variants assigned as *Silver* are considered as in-frame indels, truncation events in non-tumor suppressor genes. Variants had to be reported in at least one of the following databases: UMD, LOVD, RB1, or ALSoD [20]. The variant is assigned as *Bronze* if predicted to be tolerant by in silico algorithms.

For PeCanPie, we used the union of their internal set of cancer predisposition genes and the Cancer Gene Census v94 from COSMIC [21] containing 712 and 723 genes, respectively, generating a list of 862 genes.

Only gold and silver variants were considered and studied separately for further analysis and filtering. The variants with fewer than 20 total reads or variant allele frequency (VAF) less than 0.25 were discarded for further analysis.

### 2.7. Statistical Analysis

We performed a descriptive analysis to calculate central tendency and dispersion measures for quantitative variables and absolute/relative frequencies for categorical variables. We also constructed logistic regression models to analyze the predictors of *Gold* variants. The final model included age < 30 years at diagnosis, family history of cancer, immunohistochemical subtype, advanced stage (IIB–IV), and continuous BMI. To evaluate disease-free and overall survival, we performed a Kaplan–Meier analysis, stratified by *Gold* variant, and considered time (in months) from diagnosis to the date of first recurrence or death. To evaluate differences by mutational status, we performed a Log-rank test. For all tests, we assumed a *p*-value < 0.05 to determine statistical significance. We used STATA 14^®^ software to perform all statistical analysis tests.

## 3. Results

### 3.1. Clinical Population

A summary of our cohort characteristics, including 115 patients, is shown in Table 1 (raw data in Table S1). The median age was 33.9 years at diagnosis, and 18.3% were younger than 30. Patients diagnosed at advanced clinical stages (IIB–IV) corresponded to 65% of the cohort. Ductal histology (85%) and Luminal-B (50%) subtype were the most common in this group. A family history of cancer was present in 32.2% of these young women. All participants were premenopausal. The mean age at menarche was 12.34 years, 28.7% of participants were nulliparous, and the average number of children among parous was 2.1. Breastfeeding was practiced in 68.7% of parous women. Overweight and obesity was a condition present in 61.7% of the participants.

### 3.2. Variant Categorization

**Figure 1:**
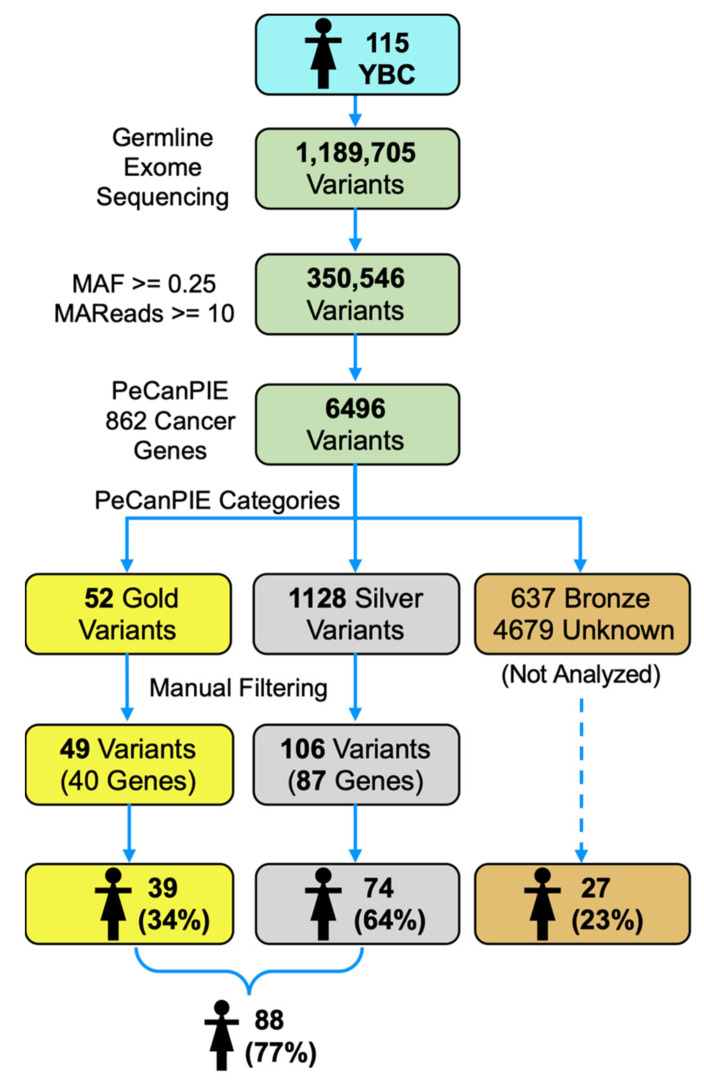
The diagram summarizes all the obtained variants with their exclusion criteria. YBC young Mexican breast cancer patients.

From the 1,189,705 raw variants, quality filtering generated 350,546 variants used for PeCanPIE annotation. PeCanPIE detected 6496 variants within the 862 cancer genes selected. After further filtering (for the number of reads described in the Methods section), 49 were categorized as *Gold* and 106 as *Silver*, corresponding to 40 and 87 genes, respectively (Figure 1). Only 39 patients (34%) showed high confidence *Gold* variants, while 74 (64%) showed *Silver* variants. Overall, 88 patients showed *Gold* or *Silver* variants.

### 3.3. High Confident Germline Variants in Cancer Genes

**Figure 2:**
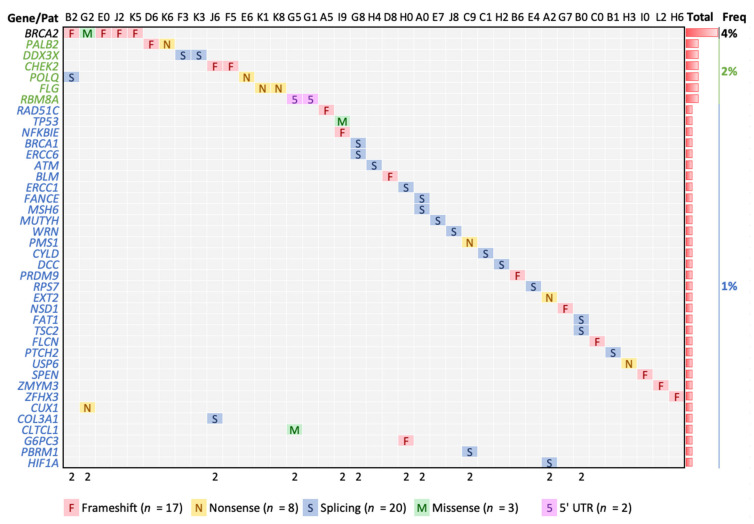
*Gold* variants detected. The top seven genes show two or more patients affected (black and green), while the rest show one patient only (blue). The 11 patients (in columns) marked with “2” show two *Gold* variants.

The criteria for *Gold* variants involve known pathogenic variants, a strong alteration variant (nonsense, frameshift, truncation) in a known pathogenic gene, and low allele frequency in public non-cancer databases [20]. From the 52 *Gold* variants obtained before manual filtering, two were removed (chr19, positions 34945343 and 34945354, *UBA2* gene) because six patients showed alternative and varied genotypes at those positions, always with fewer reads in the alternate genotype suggesting mapping and sequencing artifacts. Additionally, one variant in (chr16, position 72991715, *ZFHX3* gene) was also removed because it was present in all patients suggesting a common variant. Thus, 49 variants involving 40 genes and 39 patients were finally designated as *Gold* (Figure 2 and Table 2). All variants were heterozygous. Except for one variant present in two patients (*RBM8A*), all *Gold* variants were observed only in one patient. Of these variants, we noted 20 splicing, 17 frameshift, eight nonsense, three missense, and one ‘5-UTR. Most patients showed only one *Gold* variant, but 11 patients of 39 (28%) showed two. The well-known *BRCA2* gene was the most recurrently altered, observed in five patients, showing four frameshifts and one missense. The following most frequent alterations were observed in *CHEK2*, *PALB2*, *POLQ*, *DDX3X*, and *FLG* affecting two patients. From these genes observed in two or more patients, the variants observed in *BRCA2*, *PALB2*, and *CHEK2* were also found in ClinVar with documented association to BC (Table S2). However, variants in *POLQ*, *DDX3X*, *FLG*, and *RBM8A* are barely reported in BC and will be further described.

*DDX3X*, located in Xp11.4, encodes for an RNA helicase linked to somatic mutations in medulloblastoma [22]. It is also X chromosome inactivated in ovarian cancer [23]. Germline mutations have been reported in female brain development and disability, whose variants were observed on the *Helicase ATP-binding* and the *Helicase C-terminal* domains [24,25]. The two observed T→C heterozygous variants in our cohort affect the exon-intron splicing sites located in the *Helicase C-terminal* region (G539 and S590) responsible for the interaction with the nuclear mRNA export receptor TAP [26]. Moreover, DDX3X plays a role in DNA damage response [27]. There is no evidence in the clinical record for mental disability in these patients. These pieces of evidence suggest that DDX3X is potentially a predisposing gene in young BC patients.

*POLQ* encodes a polymerase involved in DNA repair [28,29]. Germline mutations in BC have been reported mainly in non BRCA1/2 carriers [30,31]. In our data, we observed one splicing and one nonsense variant in I2385V and L1430*, respectively. Consistent to the above studies, these *POLQ* positive patients were not carriers of BRCA1/2 or TP53 variants.

*FLG* encodes for filaggrin that aggregates keratin intermediate filaments in the mammalian epidermis. FLG germline variants have been recently reported in around 16% of Taiwanese BC patients [28] and 17% of hepatocellular carcinomas from Thailand [29]. In addition, it has been found somatically mutated in 10% of ER + BC patients [30]. We observed two G→A nonsense mutations (R788* and R501*), each one affecting a single patient. One of these patients carrying a *FLG* germline variant had a second primary contralateral breast cancer (K1).

We observed a promoter variant in *RBM8A* affecting two patients. According to ClinVar (ID 30464), this recessive variant causes a decrease in gene expression [31], which is crucial when combined with another severe variant affecting patients with Radial aplasia-thrombocytopenia syndrome. RBM8A differential expression has been noted in several cancers types but observed more expressed in the tumor than in the normal tissue [32], which seems counterintuitive. The allelic fraction reported in gnomAD is 1.8%, similar to the 1.7% observed in our sample, suggesting that promoter variants may be random. Like reports in ClinVar, it may need to be combined with another unknown alteration. Overall, the evidence is unclear, suggesting that the promoter variant in *RBM8A* is a variant of unknown significance (VUS).

From the genes present in only one patient, 12 genes have strong support shown by their reports in ClinVar for pathogenic or likely pathogenic variants in *ATM*, *BLM*, *BRCA1*, *CLTCL1*, *ERCC6*, *FANCE*, *G6PC3*, *MSH6*, *MUTYH*, *TP53*, and *TSC2*. Moreover, some of these genes also show germline mutations in BC patients. For example, *ERCC6* has also been reported in Brazilian YBC patients [33] and Lebanon familial BC [34]; *BLM* in Russian YBC patients [35] and USA patients [36]; *TSC2* in Italian patients [37]; and *ATM* is also well known in BC [11].

The remaining *Gold* variants genes are, by definition in PeCanPIE, associated with diseases in databases; inquiringly, they do not show clear evidence of pathogenicity, specifically in ClinVar. All these variants carry protein truncations in genes where the loss of function (LoF) mutations is a known mechanism of disease (*BLM*, *COL3A1*, *CUX1*, *CYLD*, *ERCC1*, *EXT2*, *FAT1*, *FLCN*, *NFKBIE*, *NSD1*, *PBRM1*, *PMS1*, *PRDM9*, *PTCH2*, *RAD51C*, *RPS7*, *USP6*, *WRN*, and *ZFHX3*). Many of these genes already show evidence of variants in BC in other populations such as *BLM* in Slavic [38] and Brazilians [39]; *ERCC1* in Brazilians [40]; *PMS1* in men [41]; *WRN* in Latins [42], or in other cancers such as *CYLD* in head and neck [43]; *EXT2* in osteochondromas and chondrosarcomas [44,45]; *FAT1* in retinoblastomas [46]; *NSD1* and *PBRM1* in renal cell carcinoma [47,48]; *PRDM9* in acute lymphoblastic leukemia [49]; *PTCH2* in rhabdomyosarcoma [50]; *RAD51C* also in breast cancer [51]; *RPS7* in hypocellular bone marrow failure [52]; *SPEN*, *USP6*, and *ZFHX3* in pancreatic adenosquamous carcinoma [53]; *TICAM1* in thyroid cancer [54]; *ZFHX3* also in endometrial cancer [55]; and *DCC* in gallbladder cancer [56].

We also noted seven genes marked with *“caution”* in PeCanPIE showing the truncation close to the C-terminal (*CUX1*, *FLCN*, *FLG*, *MSH6*, *NSD1*, *PRDM9*, and *USP6*), questioning its functional effects in the cancer context. To provide additional support for these variants, we considered the probability of LoF intolerance (pLI) provided by gnomAD [18]. Natural selection purifies highly deleterious variants, therefore, genes showing fewer than expected LoF variants in a large healthy population are seen as highly LoF intolerant, proposing association to disease when observed in an individual. Thus, pLI close to 0.9 and up to 1 are a strong indicator of LoF intolerance as recommended by gnomAD. We noted pLI = 1 in *NSD1*, strongly suggesting some contribution to disease consistent with previous evidence [47]. We also noted pLI = 1 for *CUX1*, but the stop codon gain is shown at the last protein amino acid (Q678), marked in gnomAD as *‘low confidence loss of function*’. In between, we observed pLI = 0.79 in *FLCN* and pLI = 0.77 for *NFKBIE*. Contrary, we noted pLI = 0 for *PRDM9* and *USP6*; thus, although categorized as *Gold*, these variants are less likely to be pathogenic. We also noted pLI > 0.9 for *COL3A1*, *DCC*, *DDX3X*, *PBRM1*, *RPS7*, *SPEN*, *TSC2*, *ZFHX3*, and *ZMYM3*, in which a considerable proportion of the protein is altered by a frameshift, splice, or nonsense variant. We noted that one of the patients carrying TP53 and NFKBIE presented a second primary glioblastoma (I9).

We observed that the variant allelic fraction in Latino populations, is in general, low (Table 2), validating the PeCanPie filtering. Nevertheless, few variants in the order or few per a thousand (10^−3^) could indicate a random finding due to our sample size.

We explored associations between patients carrying *Gold* variants and those not, along with clinical co-variables. The adjusted model showed an association of *Gold* variants with first- and second-family history of cancer (OR 3.21; CI 95% 1.15–8.95) and age < 30 years (OR 3.74; CI 95% 1.20–11.70). None of the tumor subtypes was associated with carrying a *Golden* variant.

We also observed that an increase in one unit of continuous BMI raises the odds of detecting a *Gold* variant in young women with breast cancer (OR 1.19; CI 95% 1.07–1.34), suggesting that BMI might be a modifier in women with *Gold* germline variants, and might reduce the age of breast cancer presentation. This phenomenon has been described previously for BRCA1/2 carriers, but our results suggest BMI could be a modifier for other genes as well [57,58,59].

We noticed that women with a *Gold* variant were diagnosed in advanced stages: IIB-IV (OR 3.21; CI 95% 1.21–8.98), suggesting that a *Gold* variant might increase breast cancer aggressiveness. It has been described that BRCA1/2 carriers have a higher risk of lymph node involvement at diagnosis [60].

**Figure 3:**
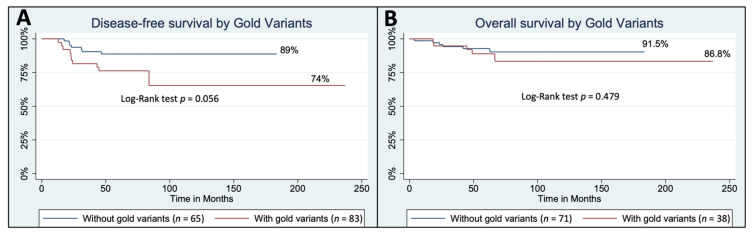
Survival analyses of YBC patients carrying *Gold* variants. (**A**) Disease free survival. (**B**) Overall survival.

The Median follow-up of this cohort was 62 months (48–73). Although not significant at *p* < 0.05 using a Log-Rank test probably due to the small sample size, we found that disease-free survival could be higher in women without *Gold* variants (89%) versus women with *Gold* variants (74%) (Figure 3A) after adjusting for clinical prognostic factors. We observed fewer differences for overall survival (OS), with 91.5% of OS in women without carrying a *Gold* variant and 86.8% OS in women with a *Gold* variant (Log-Rank test *p = 0.48*, Figure 3B). These results are consistent with many previous studies involving hereditary breast cancer and survival [61,62].

### 3.4. Modest Confidence Germline Variants

The criteria for *Silver* are in-frame indels, truncation events in non-tumor suppressor genes but associated to diseases, variants predicted to be damaging by in silico algorithms, and matches to databases such as ClinVar with fewer than two stars, BRCA Share, ALSoD, LOVD, and a locus-specific database for *APC*, *MSH2*, and *RB1*. In addition, we filtered for the 862 genes associated with cancer. Under these conditions, PeCanPIE detected 1128 *Silver* variants. To further filter *Silver* variants to choose those more likely pathogenic, we reasoned that if a variant is the same as a somatic mutation found in a cancer patient, specifically in the tumor biopsy, it could indicate a higher degree of confidence. Thus, to further explore potential pathogenic variants, we only considered *Silver* variants categorized as *Gold* or *Silver* in the PeCanPIE somatic category. This additional category considers somatic databases such as COSMIC and PCGP [63].

Thus, 106 *Silver* variants were obtained distributed in 87 genes (Table S2). Of 115 patients, 74 (64%) presented one or more silver variants, all heterozygous. Of these, 18 were missense, four splice site, one frameshift, one nonsense, six protein deletions, eight protein insertions, and 78 splice regions. Of these genes, the most frequent were *AKAP9* and *ATM* in four patients, followed by *KMT2D*, *MGA*, *COL3A1*, *NCOR2*, *ERBB2*, and *MLLT3* in three patients each. We noted the following gene families: *BRCA1/2*, *CDK 4/N2A*, *ERBB 2/3*, *ERCC 1/2/3*, *FANC A/D2/E/M*, *MRE 11/11A*, *NOTCH 1/2*, and *SMARC A4/B1/E1*. Interestingly, we detected the same missense mutation in *ERBB2* (R896H) in two patients. The gene position has been reported to activate HER2 function (R896C) [64], suggesting that R896H could affect normal function and potentially contribute to tumorigenesis.

## 4. Discussion

In the current work, we report an exome analysis of 115 young Mexican BC patients using the pipeline PeCanPIE focused on well-defined evidence of pathogenicity following ACMG guidelines. To our knowledge, this is the first effort for Mexican patients covering the germline whole exome. Previous efforts in Mexico and Latin America have focused on gene panels from 20 to 140 genes irrespective of the age of diagnosis [12,65]. Similar approaches showed a prevalence of 10.2% of pathogenic variants in BC in the USA [66]. Nevertheless, age at diagnosis is important because it may indicate an accelerated tumorigenesis process supported by recent reports showing an increase in BC incidence in young women [1,2,67,68,69,70]. Therefore, we focused on extreme phenotypes patients, where BC was diagnosed at 40 years old or less. We found that 39 patients (34%) showed a likely pathogenic *Gold* variant in 40 genes. This finding is higher than recent prevalence estimations in Latin American countries (13–25%) [42], which is likely due to our increased analysis in over the more than 800 genes by whole exome, and a higher genetic risk background of the younger population.

Comparing our *Gold* variants results with those of Quezada-Urban et al. in Mexican’s BC, where more than 53% were older than 40 years old and using a panel of 143 genes [12], only six genes were overlapping (*BRCA1/2*, *ATM*, *WRN*, *RAD51C*, and *CHEK2*). We did not find variants in 15 of the 21 genes reported (*MSR1*, *ERCC3* [1 Silver], *LIG4*, *PDE11A*, *ATR*, *FANC(I/B/C/L/M)*, *RECQL4*, *SDHB*, *MLH1*, *NBN*, and *PTEN*), and we found 36 other genes which were not present in the Quezada-Urban et al. study. Some genes, however, showed similar gene families, such as *ERCC1/6* and *FANCE*. We also noted that many genes were not reported in BC for Latin-American countries (Table 2).

In our sample, we noted that 58% of the patients were overweight at the time of diagnosis, which is consistent with the 60% reported by Quezada-Urban et al. [12] and other reports in Mexico [2]. Weight loss in the young woman has been associated with lower cancer risk in BRCA1 carriers [71]. Thus, we explored possible associations of known variants (*Gold*) to BMI. We observed a small but significant increase in BMI among *Gold* variant carriers. These results should be confirmed in larger cohorts.

We noted many *Gold* variants in genes not previously reported in Latin American cohorts but reported in BC in the USA, Europe, Asia, or other cancer types and gene families. For instance, Fanconi anemia and excision repair genes (*FANC* and *ERCC* genes) have been reported in Latin American BC cases [11]. These findings highlight the use of a broader set of genes combined with powerful analysis tools, to expand the results.

PeCanPIE uses a pipeline considering the observed variant frequency among ‘healthy’ populations deposited in databases such as ExAC, which is primarily based on Caucasian populations [72]. In the *Silver* category, we noted few variants present in many individuals in our cohort even with low allelic fraction in ExAC, confirming that estimations of Latin America variations are needed to identify common variants in this population. Although PeCanPIE was initially conceived for pediatric cancers, they included several gene databases from other cancers reaching 712 genes. To complement this, we added 723 genes from COSMIC v94. Thus, our analysis was limited to the unified 862 genes from these two sources. If we extend the analysis to the whole exome and focus on *Gold* variants, besides the two common variants that would need to be removed, six genes are added (*C8B*, *DMD*, *HBB*, *IRF8*, *KCNQ1*, and *MYBPC3*, of which three are splices, two frameshifts, and one nonsense). Nevertheless, these were not considered in our analysis, since we focus on more likely genes with a stronger background in the cancer context.

We focused on *Gold* variants because pathogenicity is theoretically the highest provided by PeCanPIE. Nevertheless, *Silver* variants may also show interesting results, such as that mentioned for HER2 (*ERBB2* R896H) and other gene families such as *CDK*, *BRCA1/2*, *ERBB*, *ERCC*, and *FANC*. However, more careful revision is needed for *Silver* variants. For example, we noted a missense mutation in five patients in *WRN* (R834C) that has been shown to abolish important WRN function [73]. Although the variant was filtered out because of quality criteria (fewer than 10 reads), this polymorphism is frequent in the Mexican and is also unlikely to be pathogenic [74]. Thus, *Silver* variants should be handled more thoroughly. This evidence also highlights that annotation tools are crucial to facilitate interpretation but that results must be revised, and tools should be continuously updated.

Overall, our study provides candidate pathogenic variants in Mexican YBC, a barely studied population. Some variants need more careful analyses; for example, those regarding splice site variants and those in *RBM8A*. In addition, recent evidence rises questions even for well-known breast cancer genes [75]. Thus, confirmatory information may be needed either by specific experimental assays or analyses of large cohorts to potentially translate our findings into clinical practice.

## 5. Conclusions

We conclude that using whole exome sequencing to analyze an extended set of cancer genes, and a rigorous bioinformatic pipeline that includes PeCanPIE, we were able to identify candidate pathogenic genes for a more extensive set in young, Mexican breast cancer patients.

## Figures and Tables

**Table 1:** Clinical data of 115 BC young patients.

Clinical Data		*n*	Frequency (%)
Patients	*n*	115	
Age	Mean	33.9	
	Interquartile range	31–38	
Age at menarche	Mean	12.34	
	Interquartile range	11–13	
Parity	Nulliparous	33	28.7
	1 child	27	23.2
	2 children	32	23.5
	>3 children	23	20.0
Breastfeeding	No	37	32.2
	Yes	78	67.8
	Luminal B (Her2-positive)	19	13.2
	Luminal B (Her2-negative)	31	28.3
	Her2-positive (non-luminal)	8	6.6
	Triple Negative	30	28.3
Histology	Ductal	98	85.2
	Lobular	6	5.2
	Mixed	9	7.8
	Others	2	1.7
Clinical Stage	I	15	13.0
	II	45	39.1
	III	49	42.6
	IV	6	5.2
Consumption of Hormonal contraceptives	No	50	43.5
	Yes	65	56.55
First-grade family history of cancer	No	90	78.3
	Yes	25	21.7
Second-grade family history of cancer	No	78	67.8
	Yes	37	32.2
Body Mass Index	Normal (BMI < 25)	44	38.3
	Overweight	45	39.1
	Obesity (BMI > 30)	26	22.6

**Table 2:** Gold variants.

Chr	Position	Ref *	Alt *	Depths	Gene	LA ^+^	Type	AA Chg	Pat	pLI	AF Lat ^§^	ProtSize
11	108175579	G	A	21,19	*ATM*		Splice	E1892_E37	H4	0	1 × 10^−4^	3056
15	91341429	A	AG	15,25	*BLM*		Frameshift	T1074fs	D8	0	-	1417
17	41258472	C	A	54,35	*BRCA1*		Splice	R71_E4	G8	0	3 × 10^−5^	1863
13	32914033	CA	C	18,20	***BRCA2***		Frameshift	S1848fs	E0	0	3 × 10^−5^	3418
13	32914122	AC	A	52,30	***BRCA2***		Frameshift	N1877fs	J2	0	3 × 10^−5^	3418
13	32930653	C	CA	15,17	***BRCA2***		Frameshift	S2509fs	B2	0	-	3418
13	32937507	A	G	44,30	***BRCA2***		Missense	D2723G	G2	0	A:C 0	3418
13	32954260	CG	C	23,17	***BRCA2***		Frameshift	V3079fs	K5	0	2 × 10^−4^	3418
22	29091718	TA	T	29,23	***CHEK2***		Frameshift	L413fs	J6	0	-	543
22	29115401	A	ATGAT	28,16	***CHEK2***		Frameshift	M222fs	F5	0	1 × 10^−4^	543
22	19222211	C	T	33,36	*CLTCL1*	No	Missense	E330K	G5	0	9 × 10^−4^	1640
2	189868190	T	A	35,26	*COL3A1*	No	Splice	P869P	J6	1	9 × 10^−5^	1466
7	101926377	C	T	57,41	*CUX1*	No	Nonsense	Q678 *	G2	1	3 × 10^−3^	678
16	50818362	T	A	30,39	*CYLD*	No	Splice	I650_E13	C1	1	3 × 10^−5^	956
18	50734187	G	A	28,23	*DCC*	No	Splice	V621_E11	H2	0.99	2 × 10^−4^	1447
X	41206109	T	C	46,39	***DDX3X***	No	Splice	G539_E15	K3	1	4 × 10^−5^	662
X	41206562	T	C	44,41	***DDX3X***	No	Splice	S590_E16	F3	1	2 × 10^−3^	662
19	45917294	T	C	21,22	*ERCC1*		Splice	V235_E7	H0	0	6 × 10^−5^	297
10	50680422	C	T	16,25	*ERCC6*	No	Splice	R975_E16	G8	0	C:A 3 × 10^−5^	1493
11	44228353	G	A	33,34	*EXT2*	No	Nonsense	W535 *	A2	0	1 × 10^−4^	718
6	35425330	C	T	25,34	*FANCE*	No	Splice	D286_E3	A0	0	3 × 10^−4^	536
4	187530955	C	G	34,21	*FAT1*	No	Splice	T3356T	B0	0	0	4588
17	17117000	CG	C	45,40	*FLCN*	No	Frameshift	R570fs	C0	0.79	C:T 3 × 10^−5^	579
1	152285000	G	A	46,43	***FLG***	No	Nonsense	R788 *	K8	0	6 × 10^−4^	4061
1	152285861	G	A	45,34	***FLG***	No	Nonsense	R501 *	K1	0	4 × 10^−3^	4061
17	42148542	TC	T	11,14	*G6PC3*	No	Frameshift	I70fs	H0	0	8 × 10^−4^	346
14	62203827	G	A	20,20	*HIF1A*	No	Splice	D417_E9	A2	0	2 × 10^−4^	826
2	48033791	GT(26)	G	15,12	*MSH6*		Splice	R1334_E9	A0	0	1 × 10^−4 &^	1360
1	45797228	C	T	25,23	*MUTYH*		Splice	G396_E13	E7	0	3 × 10^−3^	546
6	44233331	G	GC	21,12	*NFKBIE*	No	Frameshift	A57fs	I9	0.77	3 × 10^−4^	500
5	176722446	TC(6)	T	27,25	*NSD1*	No	Frameshift	S2424fs	G7	1	-	2696
16	23641062	CAG	C	25,39	***PALB2***		Frameshift	S804fs	D6	0	1 × 10^−4^	1186
16	23641139	G	C	24,36	***PALB2***		Nonsense	S779 *	K6	0	9 × 10^−5^	1186
3	52620706	TG	T	15,12	*PBRM1*	No	Splice	E1017_E21	C9	1	0 ^&^	1689
2	190728500	C	T	26,29	*PMS1*	No	Nonsense	R630 *	C9	0	1 × 10^−4^	932
3	121168273	T	C	19,22	***POLQ***		Splice	I2385V	B2	0	-	2590
3	121207489	A	T	20,15	***POLQ***		Nonsense	L1430 *	E6	0	9 × 10^−5^	2590
5	23527845	CA	C	31,42	*PRDM9*	No	Frameshift	T883fs	B6	0	6 × 10^−5^	894
1	45294985	C	T	14,13	*PTCH2*	No	Splice	L406_E10	B1	0	3 × 10^−5^	1203
17	56774167	C	CT	47,59	*RAD51C*		Frameshift	A173fs	A5	0	-	376
1	145507646	G	A	15,27	***RBM8A***	No	UTR_5	E1_UTR_5	G1	0.57	1 × 10^−2^	174
1	145507646	G	A	18,24	***RBM8A***	No	UTR_5	E1_UTR_5	G5	0.57	1 × 10^−2^	174
2	3623181	G	A	51,58	*RPS7*	No	Splice		E4	0.95	-	194
1	16262459	G	GC(27)	27,17	*SPEN*	No	Frameshift	A3242fs	I0	1	9 × 10^−4 &^	3664
17	7578406	C	T	22,29	*TP53*		Missense	R175H	I9	0.53	0	393
16	2124201	C	T	44,31	*TSC2*	No	Splice	R786C	B0	1	0	1807
17	5074956	T	A	83,62	*USP6*	No	Nonsense	Y1343 *	H3	0	9 × 10^−5^	1406
8	31014882	A	G	13,13	*WRN*		Splice	K1274_E33	J8	0	1 × 10^−4^	1432
16	72991713	C	CC(9)	20,14	*ZFHX3*	No	Frameshift	A778fs	H6	1	0	3703
X	70466308	GTGGT	G	28,11	*ZMYM3*	No	Frameshift	P821fs	L2	1	-	1370

* Numbers in parenthesis represent the total length. ^+^ Represent whether the gene has been reported in Latin-American BC patients in the Urbina-Lara et al. analysis [11]. ^§^ Allele frequency in Latino population from GnomAD website (https://gnomad.broadinstitute.org/, accessed on 1 December 2021). Variants in GnomAD slightly different to those found are explicitly indicated or marked with ^&^. A total of 50 variants is shown, 49 unique (RBM8A is present in two patients). Genomic positions correspond to hg19. Ref and Alt refer to reference and alternate alleles respectively. Depths refers to Ref and Alt alleles respectively. AA Chg refers to aminoacid change. Pat refers to patient. pLI refers to the probability of LoF intolerance. ProtSize refers to canonical transcript protein size in aminoacids. Bold genes mark those found more than once. AF Lat = Allele Frequency in Latin Americans.

## Data Availability

The data presented in this study may be available on request from the corresponding author. The data are not publicly available due to embargo period.

## Supplementary Materials

The following supporting information can be downloaded at: https://www.mdpi.com/article/10.3390/cancers14071647/s1, Table S1: Clinical Data, Table S2: Gold Variants.
